# Metatranscriptomic response of the wheat holobiont to decreasing soil water content

**DOI:** 10.1038/s43705-023-00235-7

**Published:** 2023-04-15

**Authors:** Pranav M. Pande, Hamed Azarbad, Julien Tremblay, Marc St-Arnaud, Etienne Yergeau

**Affiliations:** 1 Institut national de la recherche scientifique, Centre Armand-Frappier Santé Biotechnologie, Laval, Québec H7V 1B7 Canada; 2 Department of Biology, Evolutionary Ecology of Plants, Philipps-University Marburg, Marburg, Germany; 3 National Research Council of Canada, Energy Mining and Environment, Montréal, Québec Canada; 4 Institut de recherche en biologie végétale, Université de Montréal et Jardin Botanique de Montréal, Montréal, Québec Canada

**Keywords:** Microbial ecology, Soil microbiology

## Abstract

Crops associate with microorganisms that help their resistance to biotic stress. However, it is not clear how the different partners of this association react during exposure to stress. This knowledge is needed to target the right partners when trying to adapt crops to climate change. Here, we grew wheat in the field under rainout shelters that let through 100%, 75%, 50% and 25% of the precipitation. At the peak of the growing season, we sampled plant roots and rhizosphere, and extracted and sequenced their RNA. We compared the 100% and the 25% treatments using differential abundance analysis. In the roots, most of the differentially abundant (DA) transcripts belonged to the fungi, and most were more abundant in the 25% precipitation treatment. About 10% of the DA transcripts belonged to the plant and most were less abundant in the 25% precipitation treatment. In the rhizosphere, most of the DA transcripts belonged to the bacteria and were generally more abundant in the 25% precipitation treatment. Taken together, our results show that the transcriptomic response of the wheat holobiont to decreasing precipitation levels is stronger for the fungal and bacterial partners than for the plant.

## Introduction

Drought is one of the most significant threats to crops and will become more frequent and intense with climate change [1]. Both the plant and its microbiota respond to decreasing soil water content, which affects the fitness of the plant. However, because of a lack of studies integrating plant and microorganisms, the best targets for improving crop resistance to water stress are not clear. Many *Actinobacteria* and *Proteobacteria* can improve plant tolerance to drought- or salinity-related stresses [2–5]. Fungal endophytes can also improve plant performance under abiotic stress [6–8]. Mycorrhizal fungi can improve water use efficiency and reduce drought stress in wheat [9], oat [10], and corn [11]. Interestingly, endophytic and rhizospheric microorganisms isolated from environments prone to drought tend to confer plants with a better resistance to drought [8, 12]. Many mechanisms are involved in the enhancement of plant drought tolerance by microbes. These include modulation of plant drought stress genes [13], reduction of the stress hormone ethylene levels through degradation of its precursor 1-aminocyclopropane-1-carboxylic acid (ACC) by the bacterial enzyme ACC deaminase [2, 3], stimulation of the expression of plant genes related to osmolytes and osmoprotectants by bacterial volatile organic compounds [14] and modulation of the plant epigenetics response to drought [15]. Plants also directly respond to water stress through genetic, molecular and physiological mechanisms [16].

A host and its microbiota form an holobiont, and their combined genomes is the hologenome [17]. Although the concept has been debated [18–21], it is useful in emphasizing the role that microbial communities play in the host biology [for more details on these concepts, see 17]. The hologenome theory of evolution [22] considers the hologenome as one evolutionary unit, which provides an interesting framework for studying the adaptation of holobionts to stressful conditions. It implies that there are microbial-driven means by which holobionts can adapt to new environmental conditions [23–25]. The hologenome can change through 1) recruitment of new microbial partners from external sources, 2) amplification or reduction of the microbial partners already in place, and 3) HGT from the external communities to the microbial partners already in place. These are coherent with the mechanisms of ecological community change put forward in the theory of ecological communities [26, 27], namely 1) migration, 2) selection, 3) speciation and 4) drift. At the transcriptomic level, the microbial response can stem from two mechanisms: 1) changes in the metagenome (by the three mechanisms listed above) and 2) changes in the gene expression of the members of the community. Although these two mechanisms cannot be disentangle using metatranscriptomics, the result will be the same: a change in the genes expressed within the holobiont. For the host, the transcriptomic response is limited to shifts in gene expression. We therefore hypothesized that most of the transcriptomic response of the wheat holobiont to decreasing soil water availability will be microbial. To test this hypothesis, we grew wheat under rainout shelters that let through 25, 50, 75 or 100% of the natural precipitation. Plant roots and rhizosphere were sampled, their RNA extracted and sequenced.

## Materials and methods

### Experimental design and sampling

Four rainfall manipulation treatments were set-up in 2016 at the Armand-Frappier Santé Biotechnologie Centre (Laval, Québec, Canada) using rain-out shelters that passively let through 25%, 50%, 75%, and 100% of the natural precipitation. The rainfall exclusion treatments were performed using 2 m × 2 m rain-out shelters, which were covered with nine, six, three, or zero 2 m × 16.7 cm sheets of transparent plastic for the 25%, 50%, 75%, and 100% treatments, respectively. The rain was intercepted by the plastic sheeting and guided in a gutter and downspout and collected in 20 L buckets that were manually emptied when they were full. Two wheat genotypes were seeded under these shelters (drought sensitive, *Triticum aestivum* cv. AC Nass and drought tolerant, *Triticum turgidum spp. durum* cv. Strongfield), and the experiment was replicated over six fully randomized blocks, resulting in 48 plots (4 treatments × 2 genotypes × 6 blocks). Plots were seeded at a density of 500 seeds per m^2^ on May 18 (2016) and May 23 (2017). Seeds harvested from each of the plots were re-seeded in the exact same plot the following year. For the current manuscript, only the Strongfield cultivar was used, from which rhizosphere soil and root samples were taken on July 26, 2017. For rhizosphere sampling, a plant was randomly selected (avoiding the edge of the plots), uprooted and shaken vigorously to remove the loosely attached soil. Soil tightly adhering to roots after shaking was considered as rhizosphere soil and was collected in sterile 1.5 ml microcentrifuge tubes. After collecting the rhizosphere soil, roots were washed with distilled water, separated from the plant and placed in sterile 15 ml Falcon tubes. Collected rhizosphere soil and root samples were flash frozen in liquid nitrogen within a span of 2 minutes after uprooting the plant to maintain the RNA integrity. Tubes were stored at −80 °C until the samples were processed for RNA extraction. At sampling, we also collected a bulk soil sample from the center of each plot for soil water content measurement. We measured soil water content by weighing soils before and after drying overnight at 105 °C.

### RNA extraction and sequencing

Total RNA was extracted from 2 g of rhizosphere soil using the RNeasy PowerSoil Total RNA Kit (QIAGEN, Canada) and 0.5 g roots using RNeasy Plant Mini Kit (QIAGEN, Canada). Extracted RNA was treated with DNAse (ThermoFisher, Canada) to remove the DNA prior to sequencing. The absence of DNA was confirmed by the lack of PCR amplification using 16S rRNA gene specific primers. Total RNA was sent for Illumina HiSeq4000 2 × 100 bp pair end sequencing at the Centre d’Expertise et de Services Génome Québec (Montréal, Québec). Libraries for rhizosphere samples were created using a microbial ribosome subtraction approach to capture all microbial transcripts, whereas libraries for root samples were created using a poly-dT reverse transcription approach to focus on the plant and fungal transcripts. The raw data produced in this study was deposited in the NCBI under Bioproject accession PRJNA880647.

### Bioinformatics

The metatranscriptome sequencing of the 24 root and 24 rhizosphere samples resulted in 2639 M reads resulting in 264 giga bases which were processed together through our metatranscriptomics bioinformatics pipeline [28]. Briefly, bases at the end of reads having a quality score less than 30 were cut off (Trimmomatic v0.32) [29] and scanned for sequencing adapters contaminants reads using DUK (http://duk.sourceforge.net/) to generate quality controlled (QC) reads. QC-passed reads from each sample were co-assembled using Megahit v1.1.2 [30] with iterative kmer sizes of 31, 41, 51, 61, 71, 81, and 91 bases. Transcript prediction was performed by calling transcripts on each assembled contig using Prodigal v2.6.2 [31]. Transcripts were annotated following the JGI’s guidelines [32] including the assignment of KEGG orthologs (KO). QC-passed reads were mapped (BWA mem v0.7.15) (unpublished - http://bio-bwa.sourceforge.net) against contigs to assess quality of metatranscriptome assembly and to obtain contig abundance profiles. Alignment files in bam format were sorted by read coordinates using samtools v1.2 [33] and only properly aligned read pairs were kept for downstream steps. Each bam file (containing properly aligned paired-reads only) was analyzed for coverage of called transcripts and contigs using bedtools (v2.17.0) [34] using a custom bed file representing transcript coordinates on each contig. Only paired reads both overlapping their contig or transcript were considered for transcript counts. Coverage profiles of each sample were merged to generate an abundance matrix (rows = contig, columns = samples) for which a corresponding CPM (Counts Per Million—normalized using the TMM method) (edgeR v3.10.2) [35]. Each contig was blasted (BLASTn v2.6.0+) against NCBI’s nt database (version downloaded from NCBI’s server on January 9th 2019) and the best hit’s taxonomic identifier was used to assign a taxonomic lineage to the contig. Taxonomic summaries were performed using MicrobiomeUtils v0.9 (github.com/microbiomeutils). The metatranscriptome co-assembly, transcript abundance, read count summaries and mapping statistics and other results generated by our bioinformatic workflow are provided in the companion online Zenodo archive (10.5281/zenodo.7121038).

### Statistical analyses

All statistical analyses were performed in R version 4.1.0. [36]. Transcript differential abundance analyses between the 100% and 25% precipitation treatments were carried out using the EBTest function of the EBSeq library with a false discovery rate (FDR) of 0.05. Anovas were performed using the aov function of the stats package. The R project folder containing the R code used for data manipulation, statistical analyses, and tables and figure generation is available on our lab GitHub repository (https://github.com/le-labo-yergeau/MT_Holobiont_Wheat). The associated transcript abundance and annotation tables, the metadata, and the soil water content files used with the R code are available on Zenodo: 10.5281/zenodo.7096909.

## Results

### Soil water content (SWC)

**Fig. 1:**
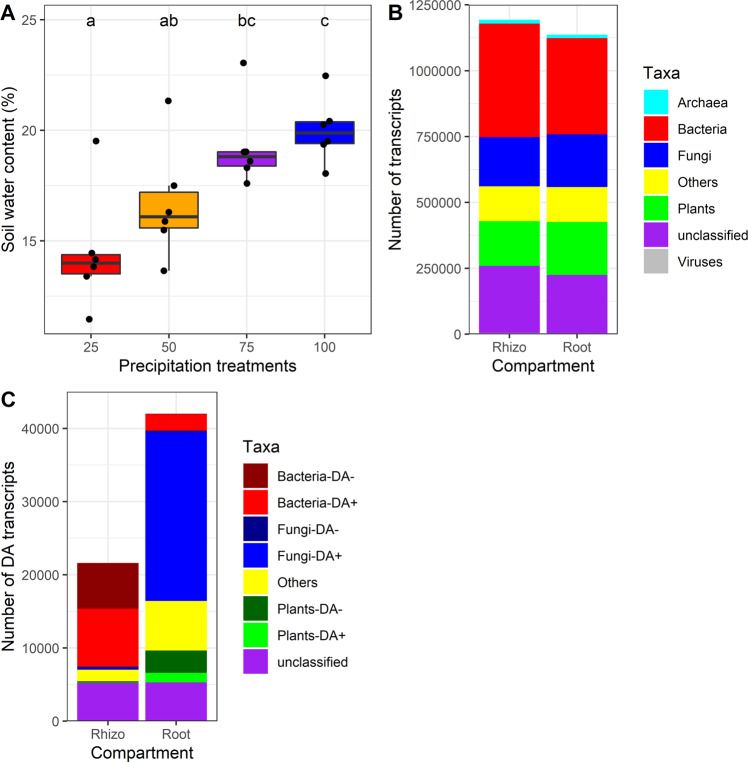
Soil water content and kingdom-level affiliation of transcripts. **A** Mean soil water content at the time of sampling for the four different precipitation manipulation treatments. **B** Kingdom-level taxonomic affiliation of the transcripts retrieved for all roots and rhizosphere samples. **C** Kingdom-level affiliation of the differentially abundant (DA) transcripts together with information if they were more or less abundant in the 25% treatment as compared to the 100% treatment.

There was a significant difference (*p* = 0.000367) between the mean SWC across the four treatments. The water content was highest in plots exposed to 100% of the natural precipitation and gradually decreased in plots receiving 75%, 50% and 25% of the natural precipitation (Fig. 1A). The SWC was of 11% at its lowest (in the 25% precipitation treatment) and of 23% at its highest (in the 100% treatment). The rest of our analyses focus on the two most extreme conditions, the 25% and 100% precipitation treatments.

### Responses of the holobiont partners

We retrieved 1,069,108,624 clean sequencing reads (per sample, mean: 22,746,992, max: 43,912,775, min: 13,943,395) that were assembled in a total of 1,269,055 transcripts, among which 1,193,501 and 1,136,587 transcripts were found in the rhizosphere soil and wheat roots, respectively. Among the wheat root transcripts, 12,792 (1.1%) belonged to the Archaea, 365,435 (32.2%) to Bacteria, 200,233 (17.6%) to Fungi, 200,823 (17.7%) to plants, 132,313 (11.6%) to other Eukaryotes, 3660 (0.3%) to viruses and 221,331 (19.5%) were not identified at the kingdom level (Fig. 1B). Among the rhizosphere soil transcripts, 14,943 (1.3%) belonged to the archaea, 430,984 (36.1%) to the bacteria, 186,745 (15.6%) to the fungi, 169,788 (14.2%) to the plants, 132,042 (11.1%) to other eukaryotes, 4255 (0.4%) to viruses, and 254,744 (21.3%) were not classified at the kingdom level (Fig. 1B).

**Fig. 2:**
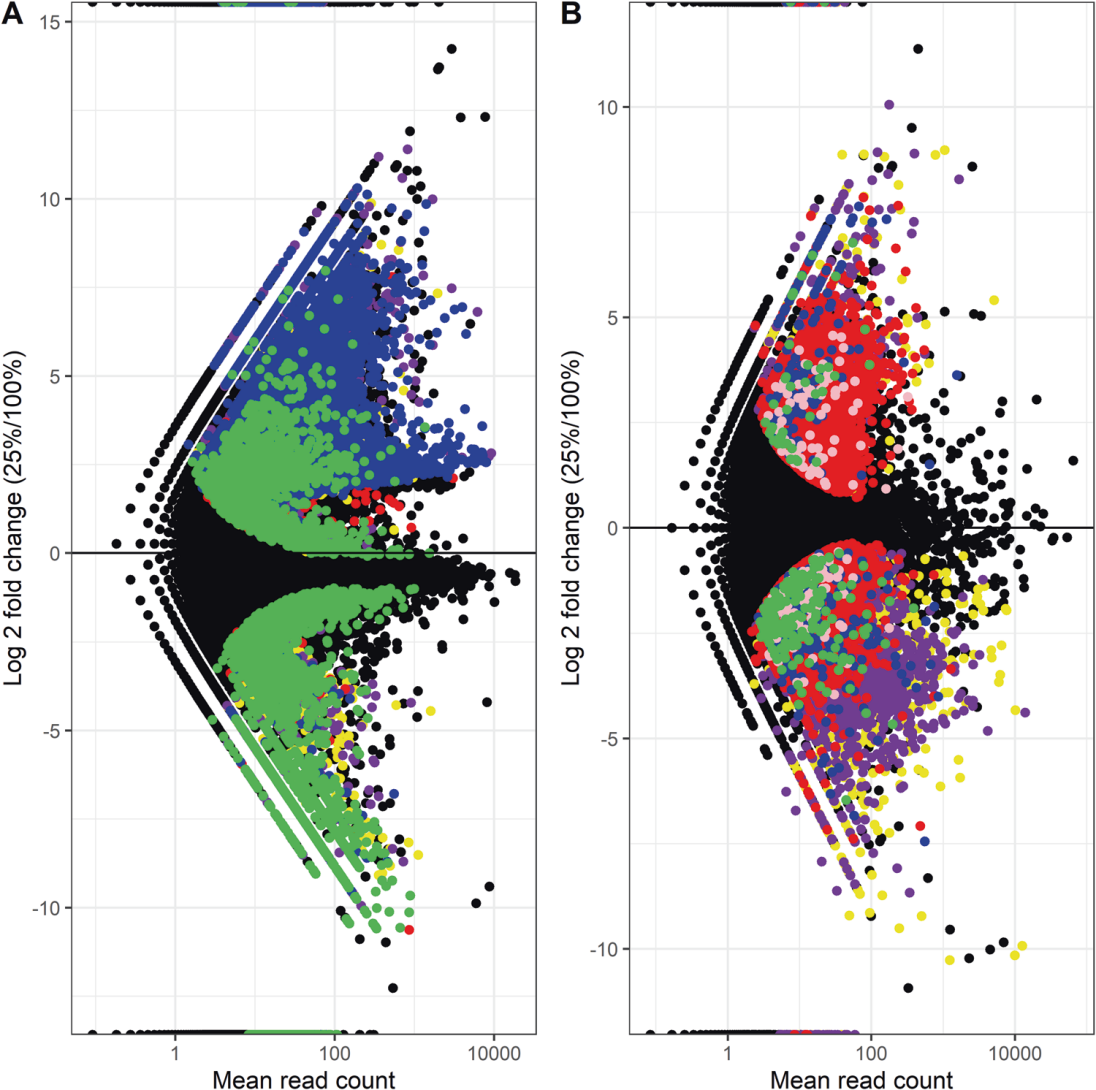
Volcano plots of transcripts. Volcano plot of transcripts log2 fold change vs. mean relative abundance, with significantly differently abundant (DA) transcripts highlighted by colors corresponding to their kingdom-level taxonomy for (**A**) roots and (**B**) rhizosphere soil. Blue: fungi, green: plant, red: bacteria, yellow: others, purple: unclassified, pink: archaea.

In the roots, among the 1,136,587 transcripts, 42,001 (3.70%) were differentially abundant (DA) at a FDR of 0.05. Among these DA transcripts, 2309 belonged to the bacteria (5.50%), 23,274 to the fungi (55.41%), 4357 to the plants (10.37%), 5303 were not classified at the kingdom level (12.63%) and 6758 belonged to other taxa (16.09%) (Figs. 1C and 2A). For bacteria and fungi, most of the DA transcripts were more abundant in the 25% treatment as compared to the 100% treatment (23,042 and 2231 more abundant vs. 232 and 78 less abundant for fungi and bacteria, respectively), whereas it was the inverse for plant (1295 genes more abundant vs. 3061 less abundant) (Figs. 1C and 2A).

In the rhizosphere, among the 1,193,501 transcripts, 21,765 (1.82%) were differentially abundant at a FDR of 0.05. Among these DA transcripts, 14,178 belonged to the bacteria (65.14%), 159 to the archaea (0.73%), 402 to the fungi (1.85%), 219 to the plants (1.01%), 5,224 were not classified at the kingdom level (24.00%) and 1,583 belonged to other taxa (7.27%) (Figs. 1C and 2B). For bacteria, slightly more DA transcripts were more abundant in the 25% treatment as compared to the 100% treatment (7,938 more abundant vs. 6,240 less abundant), whereas it was the inverse for plant (41 more abundant vs. 178 less abundant) and fungi (149 more abundant vs. 253 less abundant) (Figs. 1C and 2B).

### High level taxonomy and functions of the DA transcripts

**Fig. 3:**
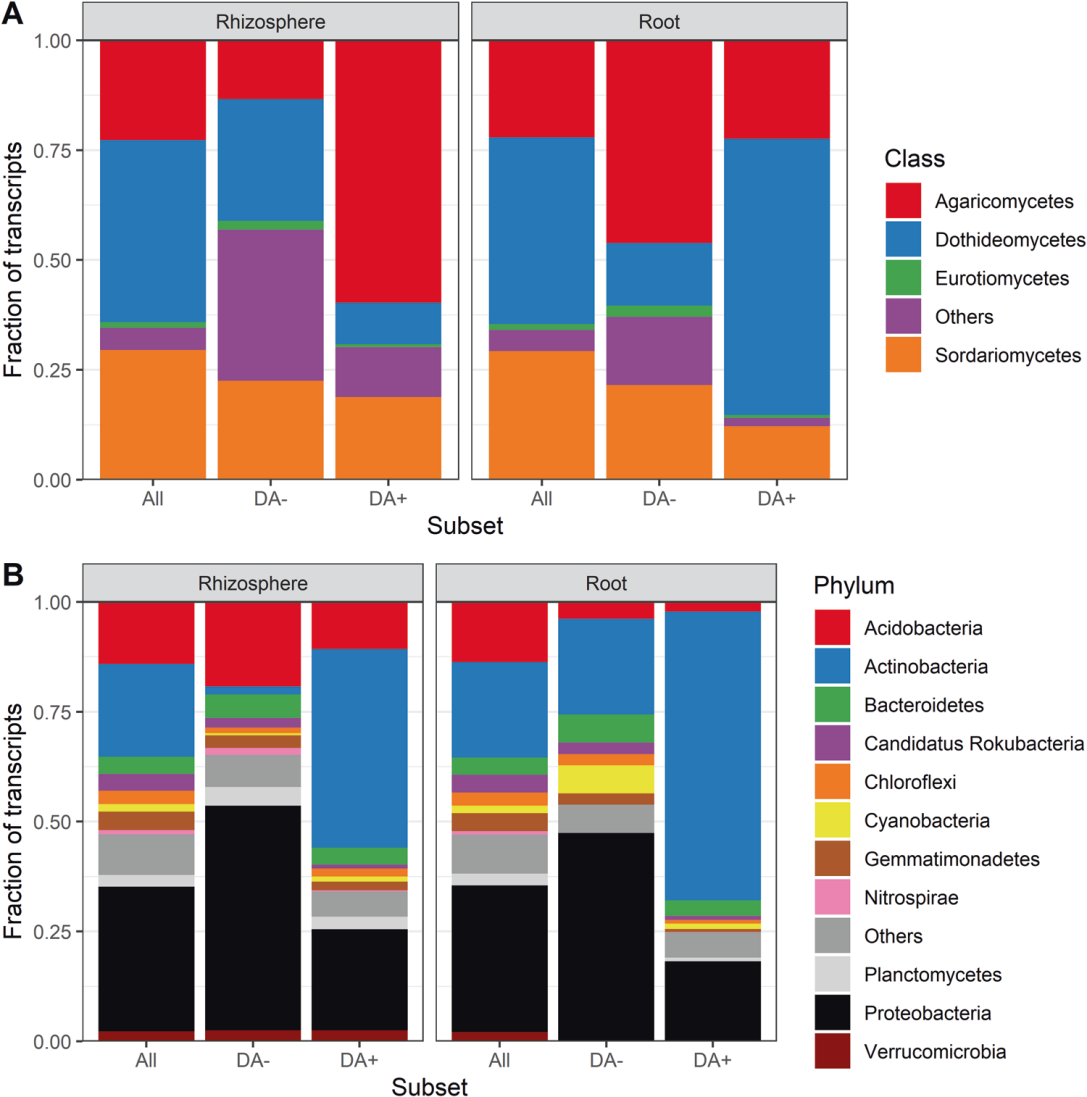
Phylum- and class-level affiliations of transcripts. Stack bar chart comparing the taxonomical affiliations of transcripts associated to (**A**) fungi and to (**B**) bacteria in the roots and the rhizosphere across all samples vs. among transcripts positively (DA+) or negatively (DA-) differentially abundant. The transcripts not classified at this level (“NULL”) were removed.

We compared the taxonomic affiliations at the phylum/class levels for all transcripts vs. the positive and negative DA transcripts in the roots and the rhizosphere (Fig. 3). Since the DA analyses result in a single list of DA transcripts per plant compartment, we are not able to test statistically for the differences in the representation of the taxa in the different subsets. However, interesting trends emerged. Some taxa were relatively less abundant among DA transcripts than among all transcripts, suggesting a lack of response to the precipitation exclusion treatments. The Sordariomycetes, Chloroflexi, Gemmatimonadetes, among others, were in this situation across all compartments, together with the Acidobacteria in the roots and the Dothideomycetes in the rhizophere (Fig. 3). Other taxa were overrepresented among the positive DA transcripts and underrepresented among the negative DA transcripts, suggesting an increase in relative abundance or an upregulation of several genes under lower soil water content. The Actinobacteria in both compartments, the Ascomycota in the rhizosphere, and the Dothideomycetes in the roots were in that situation (Fig. 3). In contrast, some taxa were overrepresented among the negative DA transcripts and underrepresented among the positive DA transcripts, suggesting a decrease in relative abundance or a downregulation of several genes under lower soil water content. The Proteobacteria, Bacteroidetes, and Eurotiomycetes in both compartments, the Acidobacteria in the rhizosphere and the Agaricomycetes in the roots showed this pattern (Fig. 3).

**Fig. 4:**
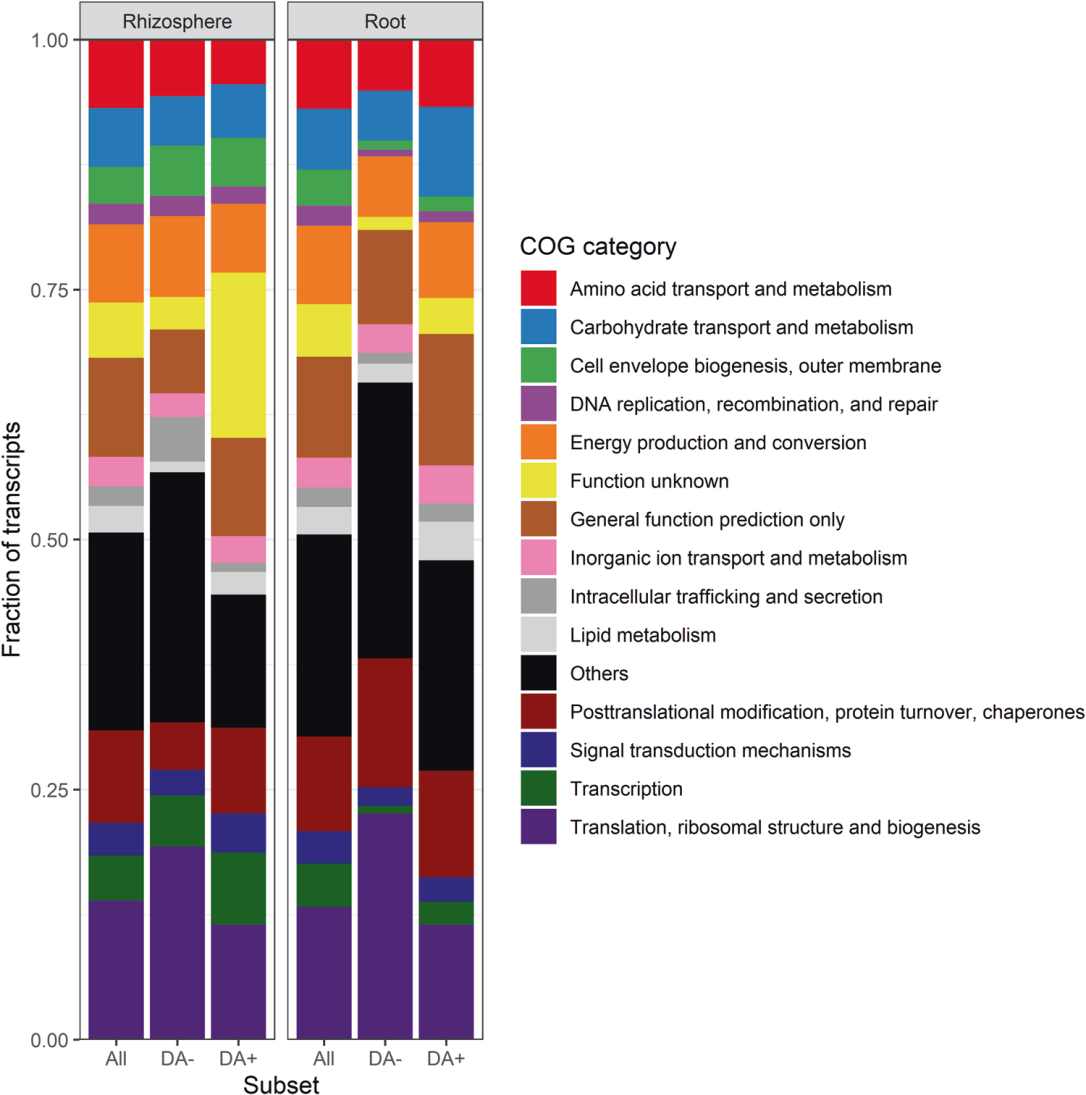
Functional affiliations of transcripts. Stack bar chart comparing the functional affiliations (COG category) of transcripts associated in (**A**) roots and (**B**) the rhizosphere across all samples vs. among transcripts positively (DA+) or negatively (DA-) differentially abundant. The transcripts not classified at this level (“NULL”) were removed.

As for COG (clusters of orthologous genes) categories, some were overrepresented in the positive DA transcripts and underrepresented in the negative DA transcripts (Fig. 4), suggesting high-level categories that are generally upregulated following a reduction of soil water content. Among these were “Carbohydrate transport and metabolism” and “Lipid metabolism” in the roots, “ “Cell envelope biogenesis, outer membrane”, “Signal transduction mechanisms”, and “Transcription” in the rhizosphere (Fig. 4). The COG categories overrepresented in the negative DA transcripts included “Translation, ribosomal structure and biogenesis” in both the rhizosphere and the roots and “Posttranslational modifications, protein turnover, chaperones” in the roots (Fig. 4). These would be COG categories that are generally downregulated with decreasing soil water content. Some COG categories were relatively less abundant among positive and negative DA transcripts than among all transcripts, suggesting a lack of response to the precipitation exclusion treatments. This included “Amino acid transport and metabolism” in the rhizosphere and “Cell envelope biogenesis, outer membrane”, “DNA replication, recombination and repair”, “Signal transduction mechanisms” and “Transcription” in the roots (Fig. 3).

### Most differentially abundant transcripts

**Fig. 5:**
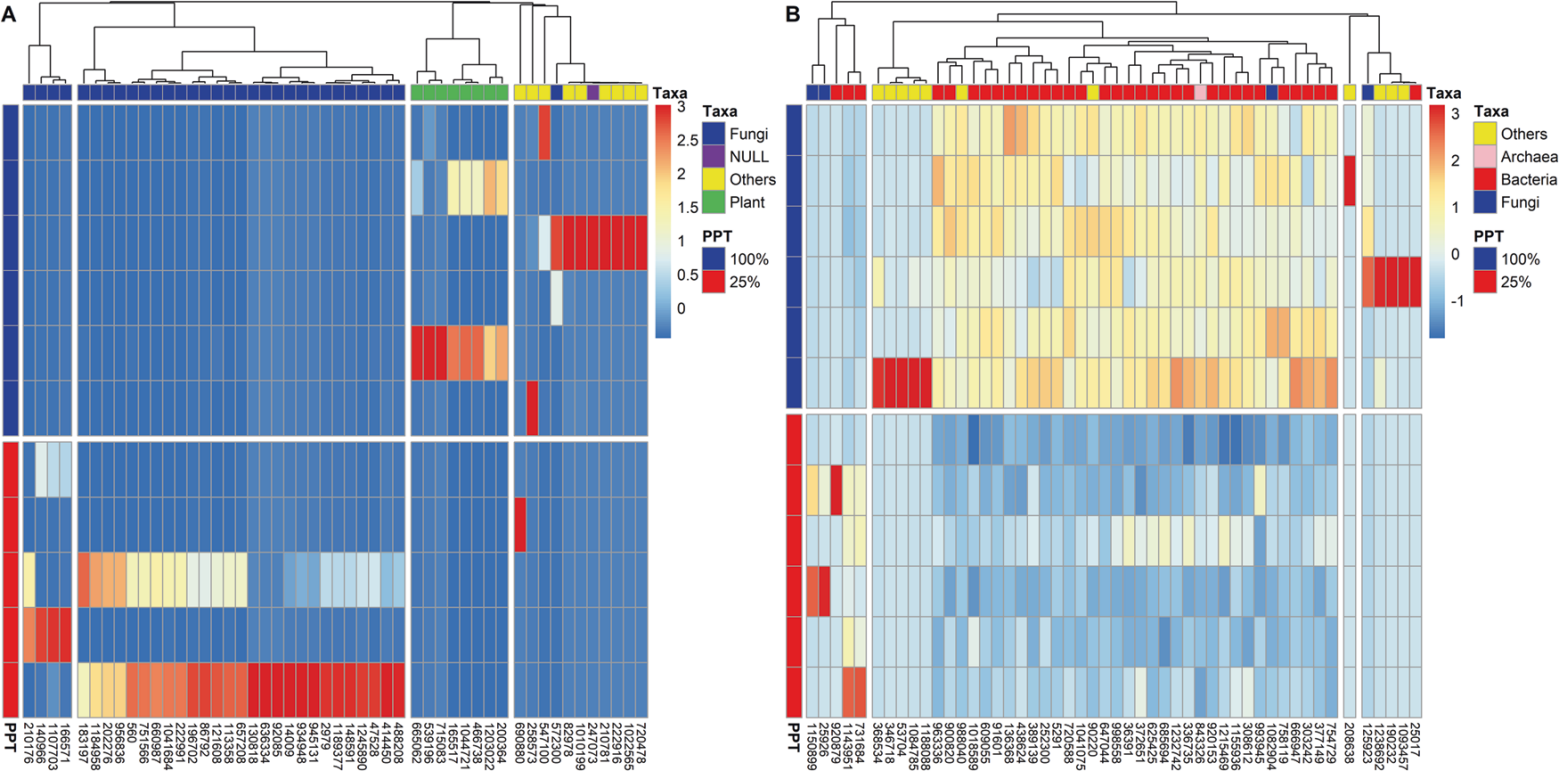
Heatmap for top DA transcripts. Heatmaps for the top 50 most differentially abundant transcripts for (**A**) roots and (**B**) rhizosphere samples.

For DA analyses of the root samples, there were many transcripts that had a P-value=0, so we sorted them by mean abundance and are showing the top 50 transcripts in Table 1 and Fig. 5A. Twenty-seven transcripts among the top 50 transcripts belonged to the *Agaricomycetes*, mostly *Coprinopsis cinerea*, and were almost all more abundant in the 25% precipitation treatment (Table 1 and Fig. 5A). Seven transcripts could be related to the wheat tribe (*Triticum aestivum* or *Aegilops tauschii*), all of which were less abundant in the 25% precipitation treatment (Table 1 and Fig. 5A). Many of the most significantly more abundant transcripts in the 25% precipitation treatment were related to amino acid and carbohydrate transport and metabolism, with transcripts such as “Amino acid transporters”, “Glycerol uptake facilitator and related permeases”, “Beta-glucanase/Beta-glucan synthetase”, “Dipeptide/tripeptide permease”, “Fucose permease”, “Neutral trehalase” and “Hexokinase” (Table 1). In contrast, many of the most significantly less abundant transcripts in the 25% precipitation treatments were linked to the COG categories “Posttranslational modification, protein turnover, chaperones” and “Secondary metabolites biosynthesis, transport and catabolism” (Table 1).

**Table 1:** Mean abundance, log2 fold change (25% vs 100% precipitation), COG function and category and taxonomic affiliation for the top 50 most abundant genes with a *P* value = 0 in the roots.

Gene id	mean	log2	*P* value	COG function	COG category	Class	Species
751566	805.00	9.57	0	Neutral trehalase	Carbohydrate transport and metabolism	*Agaricomycetes*	*Coprinopsis cinerea*
148591	759.45	8.04	0	Dipeptide/tripeptide permease	Amino acid transport and metabolism	*Agaricomycetes*	*Coprinopsis cinerea*
130818	659.18	8.17	0	Molecular chaperone, HSP90 family	Posttranslational modification, protein turnover, chaperones	*Agaricomycetes*	*Coprinopsis cinerea*
945131	441.27	8.04	0	NAD-dependent aldehyde dehydrogenases	Energy production and conversion	*Agaricomycetes*	*Hebeloma cylindrosporum*
560	359.64	7.75	0	Uncharacterized conserved protein	Function unknown	*Agaricomycetes*	*Coprinopsis cinerea*
934948	310.09	8.19	0	Hexokinase	Carbohydrate transport and metabolism	*Agaricomycetes*	*Gymnopilus dilepis*
1189377	300.36	8.14	0	Phosphatidylserine synthase	Lipid metabolism	*Agaricomycetes*	*Coprinopsis cinerea*
956836	243.82	7.64	0	Nucleoside permease	Nucleotide transport and metabolism	*Agaricomycetes*	*Coprinopsis cinerea*
121608	241.82	7.83	0	Amidases related to nicotinamidase	Secondary metabolites biosynthesis, transport, and catabolism	*Agaricomycetes*	*Coprinopsis cinerea*
92085	224.27	8.94	0	6-phosphogluconate dehydrogenase	Carbohydrate transport and metabolism	*Agaricomycetes*	*Coprinopsis cinerea*
86792	213.27	8.87	0	Phosphatidylserine/phosphatidylglycerophosphate/cardiolipin synthases and related enzymes	Lipid metabolism	*Agaricomycetes*	*Coprinopsis cinerea*
1107703	211.18	7.98	0	Polyketide synthase modules and related proteins	Secondary metabolites biosynthesis, transport, and catabolism	*Sordariomycetes*	*Stachybotrys chartarum*
2979	194.00	9.32	0	Thiamine pyrophosphate-requiring enzymes [acetolactate synthase, pyruvate dehydrogenase (cytochrome), glyoxylate carboligase, phosphonopyruvate decarboxylase]	Amino acid transport and metabolism / Coenzyme metabolism	*Agaricomycetes*	*Coprinopsis cinerea*
183197	190.18	7.70	0	Glycerol uptake facilitator and related permeases (Major Intrinsic Protein Family)	Carbohydrate transport and metabolism	*Agaricomycetes*	*Mycena chlorophos*
14009	178.73	8.61	0	NAD-dependent aldehyde dehydrogenases	Energy production and conversion	*Agaricomycetes*	*Coprinopsis cinerea*
636334	165.64	10.09	0	Predicted aminopeptidases	General function prediction only	*Agaricomycetes*	*Panaeolus cyanescens*
196702	159.00	9.03	0	Nucleoside-diphosphate-sugar epimerases	Cell envelope biogenesis, outer membrane / Carbohydrate transport and metabolism	*Agaricomycetes*	*Coprinopsis cinerea*
47528	150.45	8.37	0	Ca2+-binding actin-bundling protein fimbrin/plastin (EF-Hand superfamily)	Cytoskeleton	*Agaricomycetes*	*Coprinopsis cinerea*
657208	147.45	8.34	0	Amino acid transporters	Amino acid transport and metabolism	*Agaricomycetes*	*Dichomitus squalens*
166571	140.73	8.86	0	Predicted hydrolases or acyltransferases (alpha/beta hydrolase superfamily)	General function prediction only	*Sordariomycetes*	*Sodiomyces alkalinus*
488208	136.36	8.81	0	RNA-binding proteins (RRM domain)	General function prediction only	*Agaricomycetes*	*Coprinopsis cinerea*
222991	134.27	9.79	0	Nicotinic acid mononucleotide adenylyltransferase	Coenzyme metabolism	*Agaricomycetes*	*Coprinopsis cinerea*
165517	107.55	-Inf	0	Ca2+-binding protein (EF-Hand superfamily)	Signal transduction mechanisms / Cytoskeleton / Cell division and chromosome partitioning / General function prediction only	*Liliopsida*	*Aegilops tauschii*
210176	102.55	8.40	0	O-Glycosyl hydrolase	Cell envelope biogenesis, outer membrane	*Sordariomycetes*	*Sodiomyces alkalinus*
202276	99.73	9.36	0	Cytochrome P450	Secondary metabolites biosynthesis, transport, and catabolism	*Agaricomycetes*	*Coprinopsis cinerea*
414450	98.55	9.34	0	NAD-dependent aldehyde dehydrogenases	Energy production and conversion	*Agaricomycetes*	*Laccaria amethystina*
660987	96.09	8.30	0	Asp-tRNAAsn/Glu-tRNAGln amidotransferase A subunit and related amidases	Translation, ribosomal structure and biogenesis	*Agaricomycetes*	*Coprinopsis cinerea*
1184958	79.64	9.03	0	Beta-glucanase/Beta-glucan synthetase	Carbohydrate transport and metabolism	*Agaricomycetes*	*Coprinopsis cinerea*
572300	78.27	-Inf	0	Predicted membrane protein	Function unknown	*Agaricomycetes*	*Panaeolus cyanescens*
210781	76.73	-Inf	0	Ribosomal protein S3	Translation, ribosomal structure and biogenesis	*Magnoliopsida*	*Populus trichocarpa*
720478	74.91	-Inf	0	FKBP-type peptidyl-prolyl cis-trans isomerases 1	Posttranslational modification, protein turnover, chaperones	*Magnoliopsida*	*Populus trichocarpa*
547100	64.45	-Inf	0	Chromosome segregation ATPases	Cell division and chromosome partitioning	*Clitellata*	*Hirudo medicinalis*
466738	61.55	-Inf	0	Dehydrogenases with different specificities (related to short-chain alcohol dehydrogenases)	Secondary metabolites biosynthesis, transport, and catabolism / General function prediction only	*Liliopsida*	*Aegilops tauschii*
1041684	60.91	Inf	0	Fucose permease	Carbohydrate transport and metabolism	*Dothideomycetes*	*Ascochyta rabiei*
1022565	60.55	-Inf	0	Putative multicopper oxidases	Secondary metabolites biosynthesis, transport, and catabolism	*Magnoliopsida*	*Populus trichocarpa*
82978	60.27	-Inf	0	Ribosomal protein S19E (S16A)	Translation, ribosomal structure and biogenesis	*Magnoliopsida*	*Populus trichocarpa*
140966	58.09	Inf	0	Uncharacterized stress protein (general stress protein 26)	General function prediction only	*Sordariomycetes*	*Stachybotrys chartarum*
539196	57.45	-Inf	0	Beta-fructosidases (levanase/invertase)	Carbohydrate transport and metabolism	*Liliopsida*	*Aegilops tauschii*
665062	55.18	-Inf	0	Cytochrome P450	Secondary metabolites biosynthesis, transport, and catabolism	*Liliopsida*	*Triticum aestivum*
200364	55.00	-Inf	0	Ubiquitin-protein ligase	Posttranslational modification, protein turnover, chaperones	*Liliopsida*	*Triticum aestivum*
690880	54.64	Inf	0	Peptidyl-prolyl cis-trans isomerase (rotamase) - cyclophilin family	Posttranslational modification, protein turnover, chaperones	*Insecta*	*Onthophagus taurus*
222916	54.55	-Inf	0	Secreted trypsin-like serine protease	Posttranslational modification, protein turnover, chaperones	*Arachnida*	*AcarusAcarus siro*
1044721	52.00	-Inf	0	FOG: WD40 repeat	General function prediction only	*Liliopsida*	*Aegilops tauschii*
715083	49.45	-Inf	0	Zn-dependent oligopeptidases	Amino acid transport and metabolism	*Liliopsida*	*Aegilops tauschii*
1245890	48.91	Inf	0	Deoxyribodipyrimidine photolyase	DNA replication, recombination, and repair	*Agaricomycetes*	*Leucoagaricus sp. SymC.cos*
247073	48.91	-Inf	0	Ca2 + -binding protein (EF-Hand superfamily)	Signal transduction mechanisms / Cytoskeleton / Cell division and chromosome partitioning / General function prediction only	*NA*	*NA*
113358	48.82	Inf	0	Probable taurine catabolism dioxygenase	Secondary metabolites biosynthesis, transport, and catabolism	*Agaricomycetes*	*Coprinopsis cinerea*
258573	47.82	-Inf	0	Secreted trypsin-like serine protease	Posttranslational modification, protein turnover, chaperones	*Insecta*	*Aethina tumida*
1203022	47.00	-Inf	0	Uncharacterized conserved protein	Function unknown	*Liliopsida*	*Aegilops tauschii*
1010199	46.18	-Inf	0	Ribosomal protein HS6-type (S12/L30/L7a)	Translation, ribosomal structure and biogenesis	*Magnoliopsida*	*Populus trichocarpa*

*NA* Not Available; the transcript had no significant match to the database.

For the rhizosphere, as not that many DA transcripts had a *P*-value = 0, we are presenting the 50 lowest P-values observed in Table 2 and Fig. 5B. Half the DA transcripts with the lowest *P*-values belonged to the *Proteobacteria*, mainly the *Alpha*- and *Delta*- classes (Table 2 and Fig. 5B). Many of the most significantly less abundant transcripts in the 25% precipitation treatment were linked to the COG categories “Cell motility and secretion” and “Intracellular trafficking and secretion”, with functions related to pilus, flagella and type II and VI secretion systems (Table 2). Similar to what we observed in the roots, the fungal transcripts more abundant in the 25% precipitation treatment belonged to the *Agaricomycetes* and were related to carbohydrate and amino acid transport and metabolism (e.g., “monoamine oxidase”, “hexokinase”) (Table 2).

**Table 2:** Mean abundance, log2 fold change (25% vs 100% precipitation), COG function and category and taxonomic affiliation for the top 50 microbial genes with the lowest P-values in the rhizosphere.

gene ID	mean	log2	*P* value	COG function	COG category	Class	Species
1150899	22.42	Inf	0	Monoamine oxidase	Amino acid transport and metabolism	*Agaricomycetes*	*Leucoagaricus sp.*
1238692	18.00	-Inf	0	Heme/copper-type cytochrome/quinol oxidase, subunit 3	Energy production and conversion	*Clitellata*	*Enchytraeus albidus*
190232	9.83	-Inf	0	Ferritin-like protein	Inorganic ion transport and metabolism	*Insecta*	*Sipha flava*
25017	12.17	-Inf	0	Zn-dependent protease with chaperone function	Posttranslational modification, protein turnover, chaperones	*Gammaproteobacteria*	*Buchnera aphidicola*
252300	20.67	−2.34	0	Cold shock proteins	Transcription	*Deltaproteobacteria*	*Minicystis rosea*
25926	15.83	Inf	0	Hexokinase	Carbohydrate transport and metabolism	*Agaricomycetes*	*Gymnopilus dilepis*
346718	25.00	-Inf	0	Myosin heavy chain	Cytoskeleton	*Clitellata*	*Helobdella robusta*
368534	11.33	-Inf	0	Ribosomal protein L20A (L18A)	Translation, ribosomal structure and biogenesis	*Clitellata*	*Helobdella robusta*
53704	38.00	-Inf	0	Cytochrome b subunit of the bc complex	Energy production and conversion	*Clitellata*	*Enchytraeus cf. crypticus*
685694	25.67	−1.87	0	Phage tail sheath protein FI	General function prediction only	*Deltaproteobacteria*	*Haliangium ochraceum*
90220	31.67	−2.15	0	Actin and related proteins	Cytoskeleton	*NA*	*Dictyostelium discoideum*
125923	554.00	−7.44	6.66E-16	Putative intracellular protease/amidase	General function prediction only	*Microbotryomycetes*	*Rhodotorula graminis*
625425	18.00	−2.05	1.89E-15	Uncharacterized protein conserved in bacteria	Function unknown	*Deltaproteobacteria*	*Sorangium cellulosum*
1093457	8.67	-Inf	3.11E-15	Glutathione S-transferase	Posttranslational modification, protein turnover, chaperones	*Insecta*	*Aphis citricidus*
900820	41.58	−1.77	3.55E-15	Predicted permease	General function prediction only	*NA*	*Verrucomicrobia bacterium*
988040	16.50	−3.51	5.00E-15	Translation elongation factor EF-1alpha (GTPase)	Translation, ribosomal structure and biogenesis	*NA*	*Stramenopile sp.*
438624	22.83	−1.49	1.17E-14	Outer membrane protein (porin)	Cell envelope biogenesis, outer membrane	*Betaproteobacteria*	*Betaproteobacteria bacterium*
1155936	36.50	−1.21	1.35E-14	Serine/threonine protein kinase	General function prediction only / Signal transduction mechanisms / Transcription / DNA replication, recombination, and repair	*Deltaproteobacteria*	*Minicystis rosea*
843326	19.50	−2.46	1.83E-14	Response regulator containing CheY-like receiver, AAA-type ATPase, and DNA-binding domains	Signal transduction mechanisms	*NA*	*Nitrosopumilales archaeon*
963336	20.17	−2.61	7.27E-14	ABC-type sugar transport system, periplasmic component	Carbohydrate transport and metabolism	*Actinobacteria*	*Streptomyces sp.*
920879	10.00	Inf	1.98E-13	Predicted integral membrane protein	Function unknown	*Sphingobacteriia*	*Mucilaginibacter gotjawali*
1143951	141.75	1.51	2.50E-13	Molecular chaperone (small heat shock protein)	Posttranslational modification, protein turnover, chaperones	*Alphaproteobacteria*	*Skermanella aerolata*
666947	19.08	−2.73	3.36E-13	Tfp pilus assembly protein PilE	Cell motility and secretion / Intracellular trafficking and secretion	*NA*	*Candidatus Latescibacteria*
998558	36.75	−2.31	4.82E-13	Type II secretory pathway, pseudopilin PulG	Cell motility and secretion / Intracellular trafficking and secretion	*Spartobacteria*	*Chthoniobacter flavus*
308612	18.92	−1.54	5.51E-13	Carbon dioxide concentrating mechanism/carboxysome shell protein	Secondary metabolites biosynthesis, transport, and catabolism / Energy production and conversion	*Deltaproteobacteria*	*Haliangium ochraceum*
5291	7.42	−3.34	1.34E-12	Predicted component of the type VI protein secretion system	Intracellular trafficking, secretion, and vesicular transport	*Deltaproteobacteria*	*Minicystis rosea*
91601	17.67	−2.29	2.07E-12	Type II secretory pathway, pseudopilin PulG	Cell motility and secretion / Intracellular trafficking and secretion	*NA*	*Acidobacteria bacterium*
993945	45.58	−1.31	2.20E-12	Uncharacterized conserved protein	Function unknown	*Caldilineae*	*Caldilineae bacterium*
731684	22.67	1.82	4.13E-12	Putative intracellular protease/amidase	General function prediction only	*Actinobacteria*	*Micromonospora auratinigra*
920153	13.50	−1.91	4.87E-12	DNA-directed RNA polymerase, alpha subunit/40 kD subunit	Transcription	*Deltaproteobacteria*	*Haliangium sp.*
389139	16.67	−1.66	7.06E-12	Flagellin and related hook-associated proteins	Cell motility and secretion	*Alphaproteobacteria*	*Asticcacaulis taihuensis*
647044	11.50	−2.74	9.44E-12	Glutamine synthetase	Amino acid transport and metabolism	*Deltaproteobacteria*	*Geobacter sp.*
1215469	48.33	−1.06	9.81E-12	Heme/copper-type cytochrome/quinol oxidases, subunit 1	Energy production and conversion	*Deltaproteobacteria*	*Sorangiineae bacterium*
758119	14.33	−1.92	1.03E-11	Nitrogen regulatory protein PII	Amino acid transport and metabolism	*Deltaproteobacteria*	*Anaeromyxobacter dehalogenans*
1188088	7.67	-Inf	1.25E-11	Translation elongation factor EF-1alpha (GTPase)	Translation, ribosomal structure and biogenesis	*Clitellata*	*Enchytraeus sp. Enc*
372651	28.00	−1.24	1.32E-11	S-adenosylmethionine synthetase	Coenzyme metabolism	*Deltaproteobacteria*	*Labilithrix luteola*
1084785	7.67	-Inf	1.33E-11	Ribosomal protein L23	Translation, ribosomal structure and biogenesis	*Polychaeta*	*Sipunculus nudus*
377149	14.92	−2.15	1.43E-11	Biopolymer transport proteins	Intracellular trafficking and secretion	*Deltaproteobacteria*	*Deltaproteobacteria bacterium*
1018589	19.17	−1.44	2.74E-11	F0F1-type ATP synthase, alpha subunit	Energy production and conversion	*Clostridia*	*Butyricicoccus pullicaecorum*
303242	13.33	−2.98	2.76E-11	Competence protein ComGC	Intracellular trafficking and secretion	*NA*	*Acidobacteria bacterium*
1232742	49.17	−2.26	2.90E-11	Type II secretory pathway, pseudopilin PulG	Cell motility and secretion / Intracellular trafficking and secretion	*NA*	*Acidobacteria bacterium*
1041075	20.58	−1.77	3.42E-11	Cytochrome c peroxidase	Inorganic ion transport and metabolism	*NA*	*Planctomycetes bacterium*
720588	9.50	−2.61	4.18E-11	DNA-directed RNA polymerase, beta subunit/140 kD subunit	Transcription	*Deltaproteobacteria*	*Haliangium ochraceum*
208638	48.17	−8.17	5.39E-11	Subtilisin-like serine proteases	Posttranslational modification, protein turnover, chaperones	*Collembola*	*Orchesella cincta*
754729	12.83	−2.58	6.88E-11	Type VI protein secretion system component Hcp (secreted cytotoxin)	Intracellular trafficking, secretion, and vesicular transport	*Deltaproteobacteria*	*Chondromyces crocatus*
1082904	17.33	−2.68	7.54E-11	Uncharacterized conserved protein	Function unknown	*Sordariomycetes*	*Sporothrix schenckii*
136368	51.50	−1.91	7.75E-11	Type II secretory pathway, component PulD	Cell motility and secretion / Intracellular trafficking and secretion	*NA*	*Armatimonadetes bacterium*
609055	9.50	−2.23	7.95E-11	Response regulators consisting of a CheY-like receiver domain and a winged-helix DNA-binding domain	Signal transduction mechanisms / Transcription	*Deltaproteobacteria*	*Sorangium cellulosum*
336735	35.50	−1.28	9.77E-11	Uncharacterized protein conserved in bacteria	Function unknown	*Bacilli*	*Anaerobacillus isosaccharinicus*
36391	17.17	−1.53	1.05E-10	GTPases - translation elongation factors	Translation, ribosomal structure and biogenesis	*Deltaproteobacteria*	*Sorangium cellulosum*

*NA* not available; the transcript had no significant match to the database.

### DA transcripts common to roots and rhizosphere

**Fig. 6:**
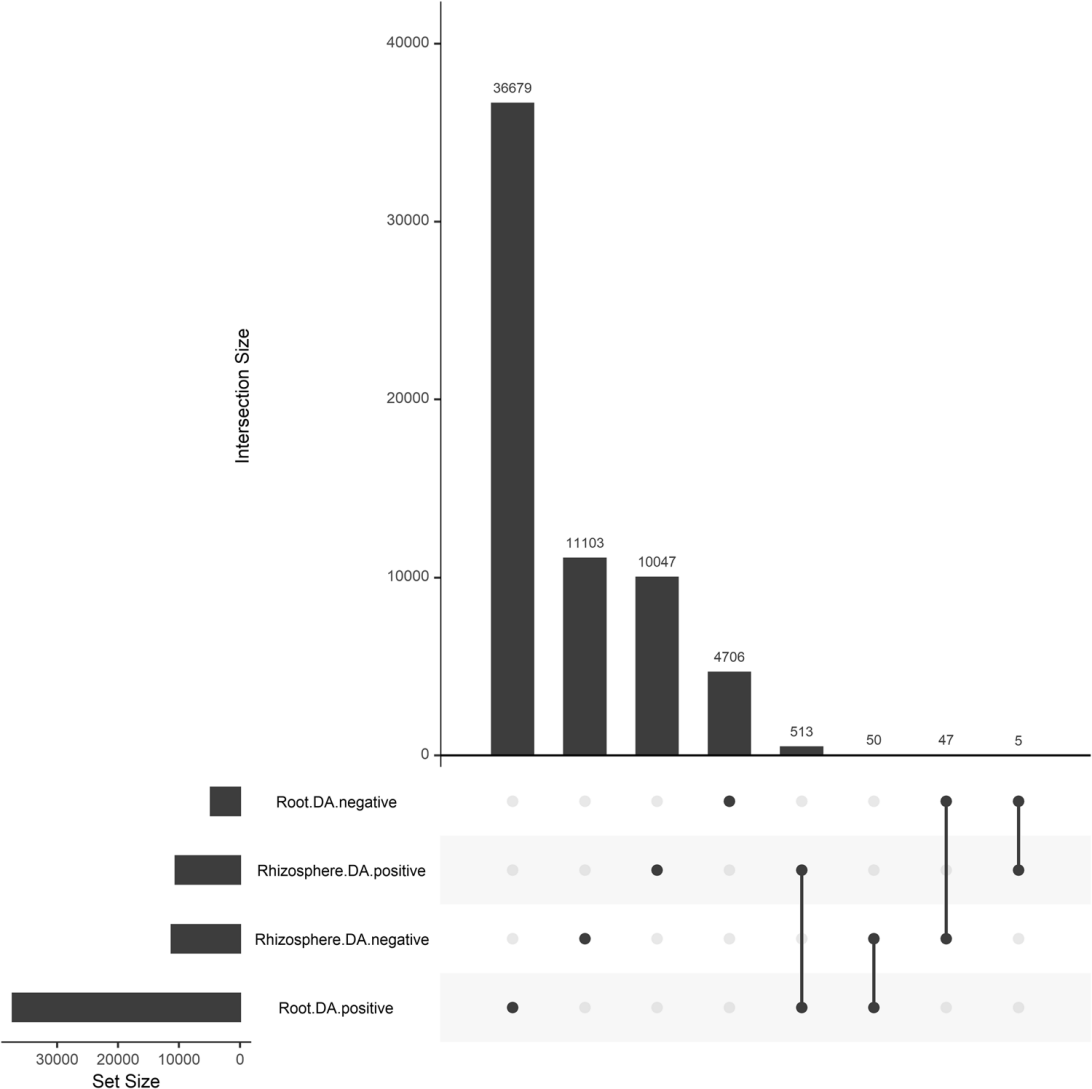
Shared and unique transcripts. Upset plot showing the shared and unique transcripts between the root and rhizosphere transcripts positively (DA+) or negatively (DA-) differentially abundant.

We looked for DA transcripts that showed a common DA response in roots and the rhizosphere. Among the 37,242 and 10,565 positive DA transcripts in roots and the rhizosphere, respectively, 513 were shared (Fig. 6). Out of these 513 transcripts, 392 were affiliated to the Actinobacteria, 27 to the Basidiomycota, 12 to the Ascomycota and 11 to the Proteobacteria (Table S1). The most represented COG category were “Translation, ribosomal structure and biogenesis” (43 transcripts), “Transcription” (33 transcripts), “Carbohydrate transport and metabolism” (29 transcripts), “Posttranslational modification, protein turnover, chaperones” (26 transcripts) and “Amino acid transport and metabolism” (14 transcripts) (Table S1). Among the 4758 and 11,200 negative DA transcripts for roots and rhizosphere, respectively, 47 transcripts were shared (Fig. 6). Most of these transcripts were not affiliated at the phylum level (26 transcripts), followed by transcripts affiliated to Streptophyta (7 transcripts) and Basidiomycota (3 transcripts) (Table S2). For COG categories, again, most of the transcripts were not affiliated with a category, and the rest were mostly affiliated to “Cytoskeleton” (5 transcripts), “Energy production and conversion” (2 transcripts), and “Translation, ribosomal structure and biogenesis” (2 transcripts) (Table S2).

## Discussion

We wanted to know how the wheat holobiont would respond to change in soil water availability at the transcriptomic level, and which of the partners would be more responsive. We were successful in reducing soil water content in a field experiment using rainout shelters, and found that, when comparing the two most contrasting treatments, most of the differentially abundant (DA) genes were linked to the fungi in the roots and to the bacteria in the rhizosphere. In the roots, most of the DA fungal transcripts were more abundant, whereas about half the DA bacterial transcripts in the rhizosphere were more abundant and the other half less abundant. These DA transcripts belonged to specific taxa and many of them could be related to genes known to help plants and microorganisms cope with water stress. Our results agree with one of our previous studies of the willow holobiont that showed that the root fungi are the strongest responders to soil contamination [37]. Bacteria responded mainly by expressing pollutant degradation genes, whereas plants did not show large transcriptomic responses [37]. Plant gene expression in the roots was more variable across plant genotypes than between contaminated and non-contaminated soils, in contrast to the strong response of bacteria to soil contamination [38].

The microbial component of the hologenome (the metagenome) is much more dynamic and plastic than the host genome [24]. Indeed, the microbial metagenome can be modified rapidly by changing the relative abundance of the community members, by recruiting new members from the environment or through mechanisms such as horizontal gene transfer (HGT) [24]. The host genome cannot be modified in response to environmental stress within a single generation. This could explain why most of the DA transcripts were microbial, as it combines changes in microbial gene expression and in the metagenome. The response of the host is limited to changing gene expression levels. The changes detected in plant gene expression could still affect important physiological processes, including root exudation [39]. As root exudates influence the transcriptome of bacteria [40], the microbial transcriptomic response to decreasing soil water content could have been mediated by the plant. The water depletion caused by our rainout shelters did not result in extremely low soil water content (around 12% soil water content at the lowest), which did not result in any visible stress on wheat. The wheat variety used is also water stress resistant, and this could explain the lack of a strong transcriptomic response. It would be interesting to contrast our results to the transcriptomic response of sensitive plant holobionts when exposed to much more extreme stress levels.

With the method used here, it is difficult to disentangle the metatranscriptomic response due to shifts in the composition of the microbial community and in the gene expression within the same community. The *Actinobacteria* well exemplify this. There was an overrepresentation of the *Actinobacteria* among positive DA transcripts in the rhizosphere, and most of the transcripts that were positively DA in both the roots and the rhizosphere were from this phylum. This phylum increases in relative abundance when soils get drier [41–45]. Inversely, the *Proteobacteria* and *Acidobacteria* were overrepresented among the negative DA transcripts and underrepresented among the positive DA transcripts, in line with their heightened sensitivity to water stress [45, 46]. In these two cases, the shifts observed are likely a combination of shifts in the relative abundance and of gene expression. Therefore, we referred to our differential expression analysis as a transcript differential abundance analysis. Looking only at high-level functional categories, like in Fig. 4, could partly solve this problem, as general trends in gene expression at this level is less likely to be influenced by shifts in community composition. Nevertheless, we argue that whatever the underlying mechanisms are, variation in the rhizosphere and root metatranscriptome complement will have functional consequences on the holobiont adaptation to stress.

Many of the most positive DA transcripts in the roots under 25% precipitation regime, were related to amino acid and carbohydrate transport and metabolism. Amino acids, such as proline, glutamine, and glycine, betaine, and carbohydrates, such as trehalose and ectoine can be used as osmolytes [47] to maintain cellular turgor and protect macromolecular structures [48]. Gram-negative bacteria produce osmolytes purely as a drought-inducible response, whereas Gram-positive bacteria tend to produce osmolytes, at least partially, on a constitutive basis [49], which could explain some of the differences in the transcriptomic response of different taxa observed here. It would be interesting to know how much this higher abundance of transcripts is beneficial to the microbes vs. the host plant. There is some evidence that microbial endophytes and rhizobacteria can increase plant osmolyte concentration [50, 51], including proline [52], and some studies have reported that microbes can exude these compounds in the plant environment [53, 54], enabling them to directly contribute to the plant osmolyte concentration during water stress. For instance, *Coprinopsis* were often reported as endophytes of plants, including *Arabidopsis* [55] and were found here among the root fungi that showed the strongest response to decreasing soil water content, with many of their more abundant transcripts related to carbohydrate or amino acid transport and metabolism.

Other important transcripts were affected by the precipitation treatments. Among the rhizosphere bacteria, transcripts related to pilus and flagella formation were less abundant with decreasing soil water content, which might be indicative of a switch from a free-living to a biofilm lifestyle. Biofilm formation is a well-known mechanism that bacteria use to cope with environmental stresses [56]. Transcripts related to heat shock proteins were more abundant in the rhizosphere and the roots under low water content, in line with their important roles for microbes and plants under water stress [57–59]. In both root and rhizosphere, there was an overrepresentation of genes related to translation among the negative DA transcripts. A similar down-regulation of the protein biosynthesis machinery was observed in a recent soil warming metatranscriptomic study [60]. The author suggested that the increased enzymatic activity and overall metabolism caused by warming could call for a lower energy investment in ribosomes, thus optimizing resource allocation [60]. In contrast, during soil drying, *Acidobacteria* and *Verrocumicrobia* reduced their ribosomal content, whereas the *Actinobacteria* increased it [61]. Similarly, among a general decrease in translation-related transcripts, we observed here that for positive DA transcripts found in both the roots and in the rhizosphere, translation-related transcripts affiliated to the Actinobacteria was the most represented category. This differential regulation of translation among microbial groups could explain the dominance of Actinobacteria under reduce soil water availability.

In conclusion, holobionts are posited to respond in a coordinated fashion to stressful events. In our case, the microbial partners were clearly the strongest responders to decreasing water content, being responsible for most of the DA transcripts across the wheat holobiont. We had hypothesized that this would be the case since transcriptomic shifts in the microbiome combines changes in the metagenome and in gene expression, something that is not possible for the host. These transcriptomic shifts were related to microbial genes and taxa, such as the Actinobacteria and osmolyte-related genes, that are known to be beneficial to plants under water stress. Because of their dynamic response and beneficial potential, the microbiome should be considered as central in efforts to adapt crop holobionts to water stress.

## Supplementary Information

Supplemental Table S1

Supplemental Table S2

## Data Availability

The raw data produced in this study was deposited in the NCBI under Bioproject accession PRJNA880647. The metatranscriptome co-assembly, gene abundance, read count summaries and mapping statistics and other results generated by our bioinformatic workflow are provided in the companion online Zenodo archive (10.5281/zenodo.7121038). The R project folder containing the R code used for data manipulation, statistical analyses, and tables and figure generation is available on our lab GitHub repository (https://github.com/le-labo-yergeau/MT_Holobiont_Wheat). The associated transcript abundance and annotation tables, the metadata, and the soil water content files used with the R code are available on Zenodo: 10.5281/zenodo.7096909.
